# Are Horses (*Equus caballus*) Sensitive to Human Emotional Cues?

**DOI:** 10.3390/ani9090630

**Published:** 2019-08-29

**Authors:** Chihiro Baba, Masahito Kawai, Ayaka Takimoto-Inose

**Affiliations:** 1 Department of Behavioral Science, Graduate School of Humanities and Human Sciences, Hokkaido University, Sapporo 060-0810, Japan; 2 Shizunai Livestock Farm, Field Science Center for Northern Biosphere, Hokkaido University, Sapporo 060-0811, Japan; 3 Center for Experimental Research in Social Sciences, Hokkaido University, Sapporo 060-0808, Japan

**Keywords:** horses, sensitivity to emotion, human–animal communication, gaze following

## Abstract

**Simple Summary:**

It is important for social animals to be sensitive to others’ emotional cues, because they can process and react to valuable social and environmental information more efficiently if they can understand others’ emotional states. Such sensitivity also seems to be adaptive in animal communication with both conspecific and heterospecific individuals, particularly for dogs and horses, because these animals have been cooperating with humans since the advent of domestication. Previous studies have demonstrated that dogs are very sensitive to human cues, such as pointing and facial or vocal expressions. However, few studies have examined whether horses exhibit a sensitivity to human emotional cues that is comparable to dogs’ sensitivity. In this study, we investigated whether horses are sensitive to human emotional cues and adjust their behavior accordingly. The results revealed that human emotional cues influenced the frequency of gaze following and the amount of time that horses looked at humans. Horses avoided following the human gaze and looked in their direction for a shorter period of time when humans displayed expressions of disgust. These findings support our hypothesis that horses exhibit sensitivity to negative human emotional cues.

**Abstract:**

Emotions are important for social animals because animals’ emotions function as beneficial cues to identify valuable resources such as food or to avoid danger by providing environmental information. Emotions also enable animals to predict individuals’ behavior and determine how to behave in a specific context. Recently, several studies have reported that dogs are highly sensitive to not only conspecific but also human emotional cues. These studies suggest that domestication may have affected such sensitivity. However, there are still few studies that examine whether other domesticated animals, in addition to dogs, exhibit sensitivity to human emotional cues. In this study, we used a gaze-following task to investigate whether horses (*Equus caballus*) are sensitive to human emotional cues (happy, neutral, disgust) and if they adjust their behavior accordingly. In the study, the experimenter suddenly turned her head to either right or left and showed emotional cues. The results revealed that horses significantly decreased the frequency with which they followed the experimenter’s gaze and the total looking time during the gaze-emotional cue presentation in the Disgust condition compared to the Neutral condition. These results suggest the possibility that horses are sensitive to human emotional cues and behave on the basis of the meaning implied by negative human emotional cues.

## 1. Introduction

Emotions aid in social animals’ survival because others’ emotions provide environmental information and allow an individual to find valuable resources or avoid threats [1,2]. Moreover, reading others’ emotional cues is necessary for animals to maintain their social bonds with group members [3], because emotional cues enable individuals to predict other individuals’ behavior and determine situation-appropriate behaviors [4]. In fact, some researchers reported that social animals such as chimpanzees (*Pan troglodytes*), dogs (*Canis familiaris*), and horses (*Equus caballus*) are sensitive to other conspecific emotional cues [5,6,7].

Sensitivity to others’ emotional cues seem to be adaptive when animals communicate not only with conspecific individuals but also with heterospecific individuals [4]. It has been reported that young chimpanzees looked referentially to their human caregiver and changed their own responses to a novel object on the basis of the caregiver’s emotional cues [8]. Furthermore, recent studies have revealed that dogs are very sensitive to human emotional cues. For example, dogs can discriminate their owner’s and unfamiliar humans’ positive facial expressions from neutral ones [9]. It is also reported that dogs can match human facial and vocal expressions that exhibit similar emotional valence as well as conspecific emotions [6] and can refer their owner’s expression when they face a novel object [10]. It is also demonstrated that dogs who were presented with two boxes choose the one that the human experimenter reacted toward with a happy expression significantly more often than the one that the experimenter reacted toward with an expression of disgust [11]. These studies suggest that dogs are very sensitive to both conspecific and human emotional cues and change their behaviors accordingly as a function of human emotional cues.

The effects of domestication on the development of sensitivity to human emotional cues has been the focus as a possible explanation for dog responses to human emotional cues in spite of their phylogenetic distance from humans [12]. Dogs are said to have been domesticated 11,000–16,000 years ago [13]. Since then, they have lived in social and anthropogenic environments with humans and might have benefited from reading human communicative cues [14]. Dogs’ ability to use human communicative cues such as pointing and eye gaze was compared with wolves’ (*Canis lupus*), the closest relative species of dogs, by the object choice task [12]. In this task, the experimenter expressed communicative cues indicating that the opaque bowl contained food, and the subjects were tested on whether they chose the bowl with food. The dogs were able to discern the correct bowl with greater frequency than the wolves. These results indicate that sensitivity to human social cues is not shared among most canids and it is peculiar to dogs. However, it has been reported that domesticated foxes (*Vulpes vulpes*) that were selected based on tameness are as responsive to human social cues as dog pups of the same ages [15]. Furthermore, the ability to use human social cues in object-choice tasks is reported in domestic horses [16] and pigs (*Sus scrofa domestica*) [17]. These studies suggest that adaptive responsiveness to various human social cues may be shared with domesticated animals. Emotional cues such as facial expression or vocalization can be potentially used by others as a form of social cues; thus, it is possible that the ability to use human emotional cues is also shared with domesticated animals. Nevertheless, there are still too few studies that examine whether domesticated animals other than dogs have this ability. Therefore, it is necessary to study whether other domestic animals are also sensitive to human emotional cues. Specifically, we considered that, in particular, animals that have been cooperating with humans in work such as hunting and farming may have developed this ability more easily because they learned to follow human social cues in order to work with humans effectively.

Horses have lived with humans as cooperative working animals for transfer and transportation for approximately 5500 years [18]. Recently, they also have played active roles in leisure and therapy as a companion animal similar to dogs. Thus, horses may have built close and cooperative relationships with humans similarly to dogs. Furthermore, horses live in a social herd intrinsically and have great social cognitive abilities. Therefore, it is considered that horses have developed sensitivity to emotional human social cues, which is supported by some studies. For example, they can use human cues to select an opaque bucket containing food in an object-choice task [16]. Horses are also able to perceive whether a person is attending to their specific needs and decide how they request food and from whom [19,20]. Furthermore, it has been reported that horses’ heart rate rose faster when they were exposed to an angry face of unfamiliar human male than when they were exposed to his smiling face [21]. This result suggests that horses distinguish angry expressions from pleasant or smiling faces and respond specifically to angry expressions. A recent study found that horses remember past facial expressions of specific people and use this emotional memory to guide future interactions [22]. Their response was more negative if they previously saw an angry photo of the person compared to a happy photo, and this memory was specific to the person whose face they saw. Moreover, it has been reported that horses are sensitive to emotional human vocal expressions and they notably show a freeze posture for significantly longer periods of time immediately following negative human vocalizations [23]. Horses perceive the emotional states of their caretakers and strangers cross-modally by associating the facial expression with the voice upon reading human emotional cues [24]. However, the neutral condition was not set in all these previous studies in horses [21,22,23,24], which suggests that horses evaluate angry human faces relatively as negative, not absolutely by comparing the basis of human neutral faces. Thus, it is possible that either horses’ evaluation of human emotional cues is biased to be positive and both human positive and negative emotional cues are generally recognized as more positive than neutral or horses’ evaluation of human emotional cues is biased to be negative and both human positive and negative emotional cues are generally recognized as more negative than neutral. By adding a neutral condition to the study, we would be able to test whether horses have the ability to absolutely evaluate human positive or negative emotional cues in comparison with the neutral condition.

In this study, we investigated whether horses are sensitive to human emotional cues (happy/neutral/disgust) using a gaze-following task. In this task, the experimenter suddenly turned her head to the right or left side and displayed an emotional cue in front of a subject horse. We used an expression of disgust rather than anger, which has been used in previous studies [21,22,23,24] to investigate whether horses are sensitive to other negative emotions. Disgust is considered to be a basic emotion that is often mixed with anger [25]. Moreover, it is natural to suddenly express disgust in many situations; thus, disgust is a suitable negative emotion to include in a gaze-following task. In a previous study of pointing-following behavior, human disgust facial expressions and voices delayed dogs’ exploration toward the baited bowl, although dogs followed the experimenter’s pointing gesture [26]. We also used a gaze-following task based on horses’ natural behavior in this study. Gaze following refers to the ability to trace an individual’s visual gaze [27]. Gaze following has been assumed to be an adaptive ability because individuals can use other gaze cues to effectively gain information about food or competition in their environment [28]. Therefore, in fact, this basic co-orienting response, gaze following, is phylogenetically widespread and is found in primates, domestic animals, and so on [29]. Horses also exhibited gaze following toward conspecifics when they see other horses showing some emotional signals in our long-term observational studies. Previous research explored long-tailed macaques’ (*Macaca fascicularis*) sensitivity to human facial expression with a gaze-following task [27]. The research suggests that gaze-following tasks may be used to reliably investigate animals’ sensitivity to human emotions.

Two behavioral measures were used in the present study: the total frequency of gaze following and the looking time in the direction toward which the experimenter shifted her gaze. In the mentioned gaze-following task study [27], long-tailed macaques changed their frequency of gaze following depending on the human experimenter’s facial expressions. Moreover, the looking time has been used as an index of horses’ attention toward conspecifics and humans [24,30,31]. Therefore, these two reactions are appropriate to measure horses’ sensitivity to human emotions. We predicted that different emotional cues displayed by a human experimenter would influence horses’ reactions if they are sensitive to human emotional cues. More concretely, we predicted that horses would follow the human experimenter’s gaze more frequently and look in that direction longer in the Happy condition than in the Neutral condition, because we assumed that when the experimenter shifts her gaze and shows positive emotion, the subject horses will willingly look in that direction. There is not enough evidence of horses’ attention to human positive emotional cues; however, in a study on dogs [11], subject dogs chose the box toward which the experimenter displayed a positive cue rather than the box toward which he displayed a neutral cue. These results indicate that dogs attend to the object evoking the experimenter’s positive emotion more frequently than to the one evoking a negative emotion. If horses are also sensitive to human emotional cues, they may attend to the place or object that the experimenter looks toward and display positive emotion. Moreover, we predicted that horses would follow the experimenter’s gaze less frequently and look in that direction for a shorter period of time when disgust was expressed, rather than neutrality. It is reported that horses experience stress as a result of human anger [21] and of conspecifics’ negative (agonistic) expressions and exhibit avoidant behaviors [7]. Therefore, we assumed that when the experimenter shifted her gaze and expressed negative cues, the horses would avoid following her gaze.

## 2. Method

### 2.1. Subjects

The subjects consisted of 14 horses (9 geldings, 5 mares; ages 3–24 years, *M* = 9.43 *SD* = 6.15) of various breeds, as described in Table 1. The horses lived at the Shizunai Livestock Farm of Field Science Center for Northern Biosphere, Hokkaido University, and they were pastured in a herd through the year. The experiment was conducted over one week at the beginning of October 2016. One horse’s data were excluded from the analysis because the experimenter showed a gaze-emotional cue when the horse was not paying attention.

### 2.2. Materials

**Figure 1:**
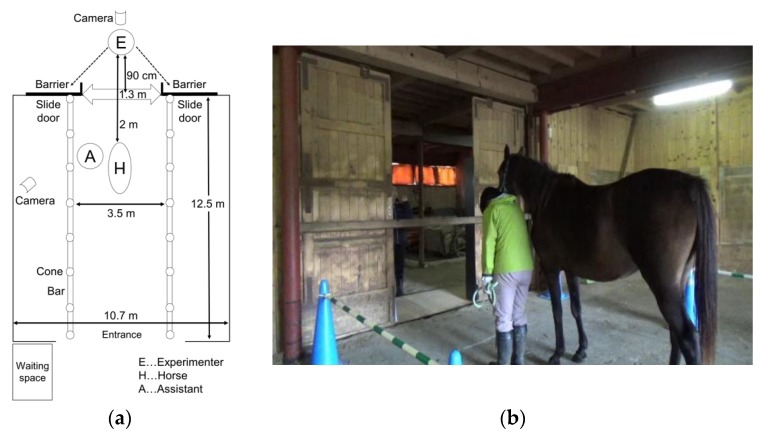
Setup for the study. (**a**) The experimental setting of the study: When the trial began, the experimenter (E) stood in front of the subject horse. When the subject horse (H) faced the experimenter, the experimenter quickly exhibited a gaze cue by turning her head to the back space of either the right or the left barrier. The dotted line shows the line of the experimenter’s visual line during the gaze-emotional cue presentations. (**b**) The photo of the experimental setting of the study: The assistant (A) guided the horse by lead to the experimental area. After the horse calmed down, the assistant looked at the floor. The assistant held the subject horse on a long- enough rope and allowed the horse to move freely within the range of the rope during the trial.

**Figure 2:**
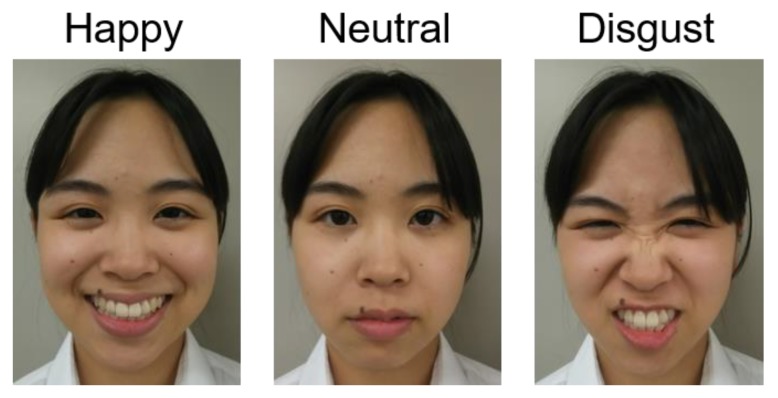
Emotional facial expressions.

The horses were tested individually in a stall of 10.7 m (W) × 12.5 m (D) × 2.7 m (H). The tests were conducted in the experimental area of the stall delimited by plastic cones, 38 cm (W) × 38 cm (D) × 68 cm (H), and bars, 190 cm (L) Figure 1a. The experimenter stood and expressed various gaze-emotional cues (Figure 2) in front of the subject between the sliding doors of the experimental area (Figure 1b). Two black opaque barriers, 22.5 cm (W) × 183 cm (H), were placed outside the sliding doors to lead the horses to believe that something behind the barriers had evoked the experimenter’s emotion. In the experiment, no object was placed behind the barriers to prevent the subject horses’ seeing the object in case they viewed the space behind the barriers, although the experimental design ensured that the horses were unable to see the space behind the barriers. In the experiments, a lead was used to control the horses, and a stopwatch (SEIKO-watch, ALBA-pico mulch-timer ADME0029, Tokyo, Japan) was used to measure the time limit. All trials were recorded on two video cameras (SONY HDR-CX670, Tokyo, Japan) attached to a tripod. One camera was located behind the experimenter and recorded the trials. The other one was located diagonally behind the horse, out of the experimental area, and recorded from the horse’s left side.

Three emotional cues were used, as in Figure 2. The experimenter’s facial expressions were based on the classical textbook of human facial emotion [25] and a previous study of sensitivity to human emotional cues in dogs [11]. As the happy emotional cue, the experimenter smiled, lowering the corner of her eyes, showing her teeth, and vocalizing “Wow!” with a high-pitched and mild voice. As the neutral emotional cue, the experimenter maintained a neutral expression and did not vocalize anything. As the disgust emotional cue, the experimenter frowned, crinkling her nose, and vocalized “Eww!” shortly with a low-pitched voice. The facial expressions and voices were validated by a third-party individual who was one of our laboratory’s members. She evaluated the three emotional cues as being appropriate by referring to the previous study on dog sensitivity to human facial and vocal expressions [11].

### 2.3. Procedure

We used a slightly modified version of the test paradigms used in the previous dog gaze-following study [32] and the effects of facial emotional expressions in a monkey gaze-following task [27]. The tests were conducted by an unfamiliar female experimenter. The experimenter waited to bend down and look down outside of the sliding doors before the trial started. The experimenter was 90 cm away from the sliding doors. An assistant waited with each subject horse in the waiting space outside the stall. The assistant guided the horse by lead to the experimental area when the experimenter gave the cue “Come in.” The assistant stopped the horse two meters away from the experimenter. After the horse calmed down, the assistant looked at the floor (Figure 1b) and gave the vocal cue “Yes” to indicate the start of a trial to the experimenter. The assistant was blind to the order of the emotional condition and the direction where the human experimenter would turn her head. The assistant held the subject horse on a long-enough rope (Figure 1b) and allowed the horse to move freely within the range of the rope during the trial. If the lope was about to be strained by the subject horse’s movement, the assistant adjusted her handling of the lead flexibly, depending on the movement of the horses’ head not to control or influence the horse’s actions. The assistant was instructed to avoid contact with the horse during the trial. When the trial began, the experimenter stood up and captured the horse’s attention by waving a hand, calling its name, and clicking. When the horse faced the experimenter, she quickly exhibited a gaze cue by turning her head to the back space of either the right or the left barrier. She displayed one of the described emotional facial expressions and matched vocalization one second after the gaze shifting and continued to display the emotional facial expression for two seconds. After expressing the gaze-emotional cues for two seconds, she displayed a neutral face. The experimenter repeated these gaze-emotional cues for another trial.

### 2.4. Experiment Design

Three experimental conditions were established as a function of emotional cues. In the Happy condition, the experimenter displayed happy emotional cues. In the Neutral condition, the experimenter displayed neutral emotional cues. In the Disgust condition, the experimenter displayed disgust emotional cues. The horses were presented with each of the three experimental conditions only once in a counter-balanced order. In each trial, the experimenter showed the same emotional cues twice. If a horse became distracted during the experiment, the experimenter captured its attention again. The trials were separated by at least 5 min. During the inter-trial interval, the horses were allowed to feed freely around the waiting space in order to relax. The direction of the experimenter’s gaze shift (right/left) was counterbalanced across all subjects.

### 2.5. Analysis

Two behavioral responses—total frequency of gaze following and total looking time—were measured between the end of the gaze shift and the moment at which the experimenter began to turn her head back to the front, which was approximately a 3 s period. “Gaze following” was defined as a horse’s action of turning its nose bridge ≤45° to the right or left of the black barrier in the same direction toward which the experimenter shifted her gaze [33]. The 45° angle was defined as the point at which the horse’s eyeball facing the barrier disappeared, with only the curve of the eye socket remaining visible and the nostril of the same side not being visible [33]. We measured the total frequency of gaze following during two gaze-emotional cue presentations per trial of each experimental condition. The maximum value was 2 in each experimental condition, because the experimenter presented gaze shift two times per trial. The “looking time” began when the horse began turning its nose bridge toward the direction of the experimenter’s gaze and ended when the horse began changing the nose bridge direction or the experimenter started to turn her head back to the front before the horse changed direction. We measured the total looking time of the horses during two gaze-emotional cue presentations per trial of each experimental condition. The maximum total looking time was 180 frames, because the experimenter presented each gaze-emotional cue for 90 frames (3 s). We analyzed the data using Friedman’s ANOVA test. Bonferroni correction was applied for the post-hoc test. We also tested the effect of the trial order on the total frequency of gaze following and the total looking time according to the condition by the Wilcoxon’s signed rank test. All video-coding was conducted by playbacking the videos frame by frame (1 frame = 1/30 s) using Cyberlink Power Director 12 (64-bit), and all statistical analyses were conducted using IBM (SPSS 22, Chicago, Illinois, USA).

The first author coded all trials from the videotapes. Another observer who was blind to the study’s purpose coded a randomly selected sample of trials (20%) to assess inter-observer reliability for the total frequency of gaze following and looking time during two gaze-emotional cue presentations. Reliability between the two coders (total frequency of gaze following: Spearman *ρ* (9) = 1.00, *p* < 0.001; total looking time: Spearman *ρ* (9) = 0.982, *p* < 0.001) was sufficient.

### 2.6. Ethical Approval

This study was approved by the Institutional Animal Care and Use Committee of Hokkaido University (Approval Number: 15-0161), and the methods were performed according to their guidelines and regulations. The caretakers of subject horses gave permission prior to their participation.

## 3. Results

### 3.1. Total Frequency of Gaze Following

**Figure 3:**
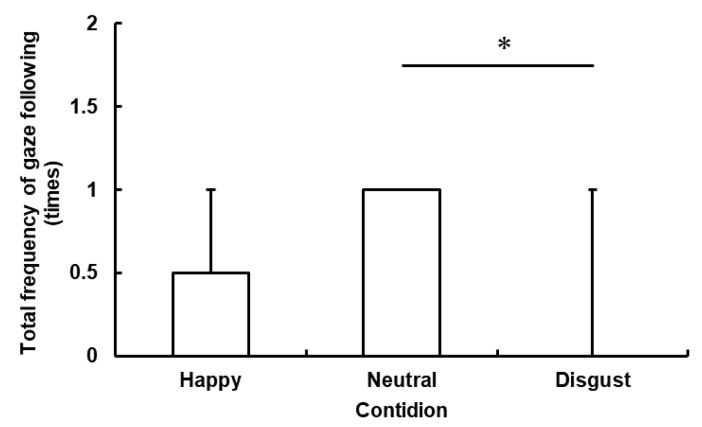
Total frequency of gaze following. Total frequency of gaze following during two gaze-emotional cue presentations per trial of each experimental condition. The maximum value was 2 in each experimental condition because the experimenter presented gaze shift two times per trial. The median in each condition was 0 in the Happy condition, 1 in the Neutral condition, and 0 in the Disgust condition (* *p* < 0.05).

The responses to emotional cues were significant (Friedman’s test: *n* = 13, *df* = 2, *χ^2^* = 8.86, *p* = 0.012), as shown in Figure 3. The result of the post-hoc comparisons revealed a significant difference only between the Neutral and the Disgust conditions (Wilcoxon’s signed rank test: *p* = 0.014). The differences were not significant between the Happy and the Neutral conditions (*p* = 0.059) and the Happy and the Disgust conditions (*p* = 0.317). This result indicated that the horses followed the experimenter’s gaze significantly less frequently in the Disgust condition than in the Neutral condition. However, there was not a significant difference between the first and the second trial in all conditions for the total frequency of gaze following (Happy: *p* = 0.564, Neutral: *p* = 1.000, Disgust: *p* = 0.157).

### 3.2. Total Looking Time

**Figure 4:**
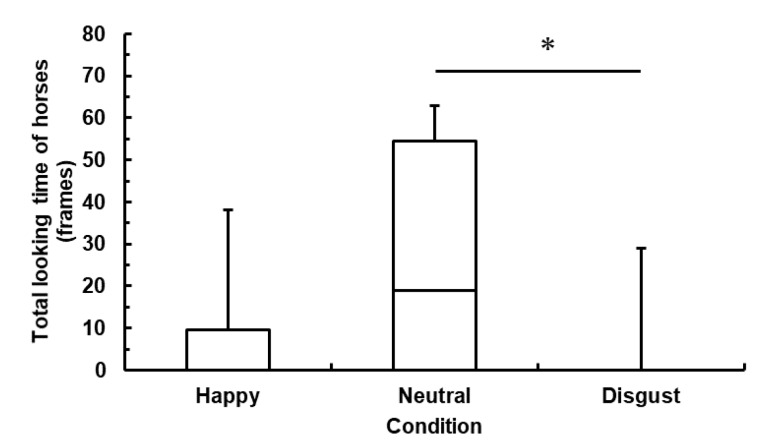
Total looking time of horses. Total looking time of the horses during two gaze-emotional cue presentations per trial of each experimental condition. The maximum total looking time was 180 frames because the experimenter presented each gaze-emotional cue for 90 frames (3 s). The median in each condition was 0 in the Happy condition, 19 in the Neutral condition, and 0 in the Disgust condition (* *p* < 0.05) (30 frames = 1 s).

The main effect of the emotional cue was significant (Friedman’s test: *n* = 13, *df* = 2, *χ^2^ =* 11.66, *p =* 0.003), as shown in Figure 4. The result of the post-hoc comparison revealed a significant difference only between the Neutral and the Disgust conditions (Wilcoxon’s signed rank test: *p* = 0.012). The differences were not significant between the Happy and the Neutral conditions (*p* = 0.038) and the Happy and the Disgust conditions (*p* = 1.00). This result indicated that the horses looked in the direction toward which the experimenter shifted her gaze for a significantly shorter time in the Disgust condition than in the Neutral condition. However, there was not a significant difference between the first and the second trial in all conditions for the total looking time of the horses (Happy: *p* = 1.000, Neutral: *p* = 0.674, Disgust: *p* = 0.180).

## 4. Discussion

In this study, we investigated whether horses are sensitive to human emotional cues using a gaze-following task. We measured horses’ behavioral responses during gaze-emotional cues, total frequency of gaze following, and total looking time. We predicted that the human experimenter’s emotional cues would influence the horses’ behavioral responses and that the total frequency of gaze following and the total looking time would change among three conditions. More concretely, the horses would follow the human experimenter’s gaze more frequently and look in the direction toward which the experimenter shifted her gaze for a longer period of time in the Happy condition than in the Neutral condition. The horses would also follow her gaze less frequently and look in the direction toward which the experimenter shifted her gaze for a shorter period of time in the Disgust condition than in the Neutral condition. As a result, human emotional cues would influence the total frequency of gaze following and total looking time in horses. Moreover, it is also suggested that horses alter their behavior according to human negative emotional cues and avoid following human gaze. In fact, we found that the horses looked in the direction toward which the experimenter expressed disgust for a shorter period of time compared to the other conditions. Therefore, our results partially support our hypotheses.

These results indicate that the total frequency of gaze following and total looking time decreased in the Disgust condition compared with the Neutral condition. One possible explanation for this phenomenon is that the emotional cue displayed by the experimenter in the Disgust condition caused the horses’ avoidance behavior. In a previous study, the horses’ heart rate rose faster when they looked at an angry human face than when they looked at a smiling human face [21]. In our study, we used an expression of disgust rather than one of anger as a negative expression, but expressions of disgust are commonly intermixed with those of anger [25]. Thus, in our study, it may be possible that horses experienced stress resulting from the disgust emotional cues and avoided it similarly to how they would react to angry expressions. Therefore, it can be deduced that horses experience stress when they are exposed to expressions of disgust and anger in humans as well as conspecific agonistic expressions, though we need further tests. Simply, horses exhibited decreased gaze following and total looking time only in the Disgust condition because they were stressed by the experimenter’s disgust emotional cue and attempted to avoid it, though we need to investigate whether horses attempt to avoid the item behind the opaque screen because it produced disgust in the human experimenter or they avoid the experimenter and her gaze because of her expression of disgust.

The differences of the total frequency of gaze following and total looking time were not significant between the Happy and the Neutral conditions. This result corresponds to a previous study’s result in which dogs showed more difficulty in distinguishing positive human emotional cues from neutral ones than in distinguishing positive human emotional cues from disgust ones [11]. There are two possible explanations for the lack of horses’ differential responses between these conditions. First, positive emotions may not incite a reaction in the subject horse. It is considered that negative emotional communication contains more important information, such as the presence of a threat, than positive communications [2]. Simply, the difference of the total frequency of gaze following and total looking time between the Happy and the Neutral conditions were not significant because responding to positive emotional cues was not urgent or necessary for the horses. Second, the particularity of the horses’ living environment may affect the results. The subject horses in this study were usually pastured in a herd and did not have many chances to interact with and share positive emotions with humans, although they had been trained for riding. Thus, they might not have been able to connect happy facial and vocal expressions with positive emotion and might not have understood the difference between happy and neutral expressions. This possibility may explain the lack of significant difference between the Happy and the Disgust conditions. To explore this possibility, horses that spend ample time in individual stalls and often interact with humans, such as those in horse-riding clubs, should be studied under the same conditions.

The results of this study support our hypothesis of horses’ sensitivity to negative human emotional cues. Specifically, they indicate that horses are capable of distinguishing human disgust cues from neutral cues and may not follow the human gaze on the basis of human expressions of disgust. The study is relevant in that it suggests the possibility that horses not only are sensitive to human emotional cues but also change in their behaviors. Moreover, we made comparisons between the positive and the neutral conditions and the negative and the neutral conditions, rather than simply comparing between the positive and the negative conditions. Thus, we found that horses evaluate human disgust as negative rather than neutral, although it is possible that the neutral cues were perceived as positive rather than neutral because there was not significant difference between the Disgust and the Happy conditions. It is likely that we could not identify significant differences between the Happy and the Disgust conditions because the horses followed the gaze in the silent condition (Neutral) and were less likely to follow the cue in conditions in which a sound was produced (Disgust and Happy). It may be beneficial to repeat this experiment with a vocal cue added in the Neutral condition. However, in this study, we set only one experimenter’s emotional cues. Therefore, so far, we cannot generalize our results unless we can replicate them with more than one human experimenters’ emotional cues and we have to discuss about our data carefully. Further testing using other methods, such as the social reference and object choice tasks, should be conducted in order to obtain more robust evidence that horses are sensitive to human emotional cues. Dogs have been reported to be able to change their behavior toward a novel object according to whether their owner exhibits a positive or a negative reaction to the object in the social reference task [10]. It has also been reported that they can use human emotional cues to select one of two alternatives in an object-choice task [11]. By using these methods, horses’ ability to use human emotional cues can be clarified. Furthermore, studies on other domesticated animals, such as cattle and sheep, and comparisons among breeds or roles in dogs or horses are necessary to reveal which factors affect their sensitivity to human emotional cues. Through these investigations, we can obtain more insight about the context that has facilitated the development of animal sensitivity to human emotional cues and whether domestication has affected this response.

## 5. Conclusions

In our study, human emotional cues influenced the frequency of horses’ gaze following and the amount of time that horses looked at humans. Horses avoided following the human gaze and looked in their direction for a shorter period of time when humans displayed expressions of disgust. The results suggest the possibility that horses are sensitive to negative human emotional cues and behave on the basis of the meaning implied by negative human emotional cues. In the future, studies on other domesticated animals, such as cattle and sheep, and comparisons among breeds or roles in dogs or horses are necessary to reveal which factors affect animals’ sensitivity to human emotional cues. Through these investigations, we can obtain more insight about the context that has facilitated the development of animal sensitivity to human emotional cues and whether domestication has affected this response.

## Figures and Tables

**Table 1:** Age, gender, and breed of the subjects.

Subjects	Age	Gender	Breed
Okaaoi 99	17	Gelding	Mixed breed of Criollo and Hokkaido
Michiyuki 99	17	Gelding	Mixed breed of Criollo and Hokkaido
Naesaka 08	8	Gelding	Hokkaido
Hikarisora	6	Mare	Mixed breed of Criollo and Hokkaido
Utaoka 10	6	Gelding	Hokkaido
Soramizu 10	6	Gelding	Hokkaido
Tachigiku	3	Gelding	Light half-breed horse
Tachimizu	3	Gelding	Light half-breed horse
Tachihime	3	Mare	Light half-breed horse
Kazeshin	24	Gelding	Light half-breed horse
Kurihime	11	Mare	Light breed horse
Anya	10	Mare	Light breed horse
Sky	9	Gelding	Appaloosa
Kurara	9	Mare	Light breed horse
